# Treatment of Obstinate Vomiting by Small Doses of Iodide of Potassium

**Published:** 1879-09

**Authors:** 


Treatment of Obstinate Vomiting by Small Doses of Iodide of Potassium.—Having noticed in the *Record* of March 15, under the above heading, an articlce from a statement made by Dr. Fornica Corsi in tbe *Gazette Obstetricale*, and having a patient suffering from obstinate and intractable vomiting arising from spinal inflammation, and having exhausted all the remedies ordinarily employed as anti-emetics, without the least amelioration in the symptoms, I determined to try the iodide in the minute doses recommended by Dr. Corsi. The vomiting had occurred immediately after taking food of any description, quantity and quality making no apparent difference. Vomiting occurred with very little effort, nausea persisting for only a short time after the contents of the stomach had been entirely rejected. This state of things had existed for at least two months, in which time she had retained only an occasional mouthful of food. After the use of injections of beef tea and egg for several days, during which time nothing but a little drink was allowed by the stomach, one or two meals were retained, but the vomiting commenced again, and continued up to the time of the administration of the iodide. I gave it in solution, in doses of 1–36, grain, repeated every hour and a half; and since then—now fourteen days—she has retained every thing she has taken, excepting oue or two meals, when she had omitted the drug for a few doses at my request, as a test.—*Medical Record.*

